# Measuring for change/Mobile Creches

**DOI:** 10.3389/fpubh.2023.1165642

**Published:** 2024-01-29

**Authors:** Chavi Vohra, Minal Shah, Atishi Mishra, Ankita Gupta

**Affiliations:** ^1^Utrecht University, Utrecht, Netherlands; ^2^Grand Challenges Canada, Victoriaville, QC, Canada; ^3^Save the Children, Kathmandu, Nepal; ^4^Mobile Creches, New Delhi, India; ^5^Agha Khan Academy, Mombasa, Kenya; ^6^Datta Meghe Institute of Higher Education & Research, Wardha, India

**Keywords:** social–emotional competencies, organisation culture, monitoring, evaluation, early childhood development, ECD

## Abstract

**Introduction:**

Research spanning decades across fields such as psychology, education, and neuroscience consistently highlights the crucial role of social–emotional skills in various aspects of personal, academic, and professional development (1–3). The fact that UNESCO recognises social–emotional learning (SEL) as essential not just for meeting its educational objectives but also for accomplishing the Sustainable Development Goals highlights the crucial role robust social–emotional development plays in establishing sustainable societies. Whilst various studies highlight the importance of SEL, there is limited attention on how organizations can contribute to building such development by consciously including SEL in their work practice.

**Process/methods:**

Our case study presents the process of integrating SEL into organizational practice over a period of 9 months. The selected constructs of SEL were determined by the organisation’s needs and values. The Measurement for Change approach was used to frame and implement the intervention, with interactive discussions being the key methodology. Data were collected via surveys, reflective sharing, and observations.

**Results:**

A shift in the selected constructs of SEL was recorded, with data highlighting individual differences.

**Conclusion:**

The process of co-design and continual reflective practice was key to achieving change within the subset of the organisation rather than the specific content of the materials used.

## Introduction

**Figure 1:**
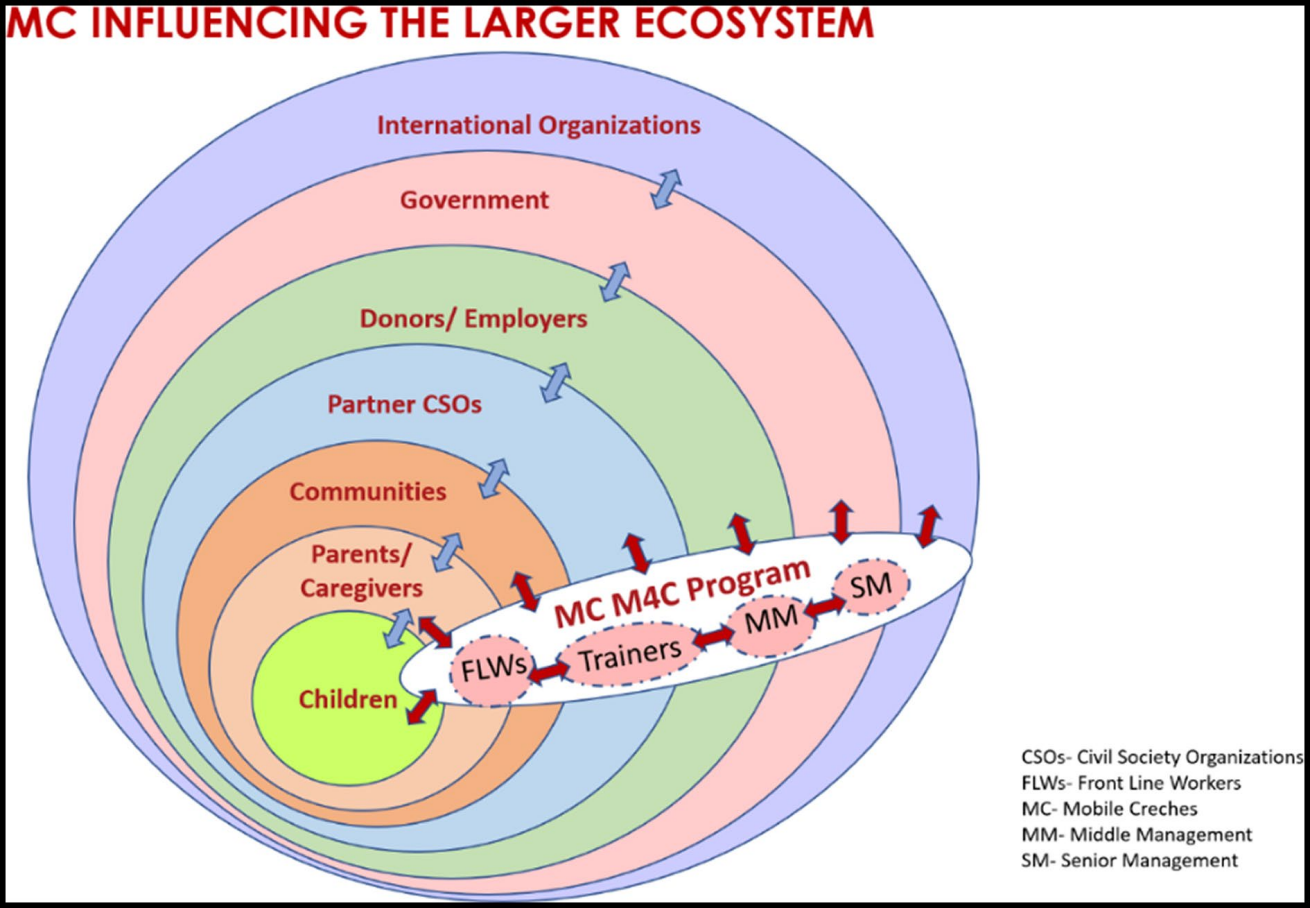
Showing how Mobile Crèches interacts with its ecosystem of various stakeholders in promoting its vision of ‘just and caring world for every child.’ The perforated boundaries and two-way arrows highlight its continual interaction with the stakeholders.

Mobile Creches (MC), with its vision for a ‘just and caring world for every child’, runs Early Child Development programmes for marginalised children aged under 8 years in India. One of MC’s latest collaborative projects led to the revision of its Early Childhood Care & Education program. The curriculum focused on strengthening and promoting social–emotional learning (SEL); MC runs training programs with parents, frontline workers, and trainers with the objective of creating a more responsive environment for the children. It was during these training programs that the participant team training the various stakeholders recognized a gap in their own social and emotional wellbeing and the programmes they were delivering. It was realized that to enhance the social–emotional competencies of children, much more must be done at the organisational level rather than just revising the children’s curriculum. This surfaced a need for creating a safe space to establish the required social–emotional support throughout the organisation [(1), p. 58–73]. As Klitkou et al. (2) highlighted, governing change toward greater sustainability requires changing the focus of intervention from incentives for changing individual behaviour to creating conditions conducive to the change of socially shared practices. Heckemann et al. [(3), p. 744–753], in their reviews, found that socio-cultural architecture, responsive carer, and strategic vision are core dimensions that characterise the emotional intelligence of nurse leadership. For our case study, these dimensions would translate into the ecosystem (see Figure 1), the core team within this case study, and its Theory of Change (Supplementary Appendix 1). Not only is EI integral in predicting individual performance but it is also core to strong leadership and success [(4), p. 9–18]. Working as a team, the core team would work toward creating a team atmosphere in which its norms would build its emotional capacity to influence other groups of people [(5), p. 80–90, 164]. Framing the Theory of Change, upskilling members within this, and emphasising their participation through shared responsibility in the process aim to strengthen the development of the organisation (6).

The dialog around adopting an approach sensitive to SEL and an emphasis on upskilling educators on SEL structured pedagogy has gained attention in the last few years. Integrating SEL into the list of dynamic Sustainable Development Goals by UNESCO has also pushed for such conversations and efforts [(7), p. 54–60]. There are an increasing number of studies underlining the importance of providing a socio-emotional conducive environment from early childhood and into the workplace, as well as calling for educators to possess this competency [(8), p. 250–262]. However, there has been little focus on how organisations can consciously include SEL in their work practice to model its importance when working with children.

This study highlights the relevance of incorporating socio-emotional coaching within the entire ecosystem around children, an ecosystem inclusive of all the stakeholders within and outside of the organisation, as seen in Figure 1. The case study presented focuses on an SEL-focused intervention trialled within a subset of the MC organisation. The outcome of this would then inform the next phase of the intervention in building the social and emotional competencies of an extended subset of the organisation. MC has national and state offices. This study took place in MC’s national office in New Delhi, India.

## Context

MC’s key programmes include childcare services, both directly and through partnering with civil society organisations and the state government, enhancing community engagement, and delivering parenting programmes. Through its extensive networking with local, national, and international organisations, MC also advocates for child-centred policies with the government. MC has reached out to 870,000 children, mainstreamed 100,000 children in formal schools, trained 6,500 women as childcare workers, run 800 day-care centres, partnered with over 250 builders, networked with over 100 non-government organisations (NGOs) and influenced key policies, laws, and programmes. Currently, MC has 150 members.

## Key programmatic elements

In developing the programme, MC has partnered with Saving Brains, a knowledge platform of Grand Challenges Canada, and worked with subject experts. Through a series of reflective and collaborative practices, MC created a framework based on the social–emotional needs of the organisation and its programmes. The goal was to promote practice and an environment that fostered the social and emotional wellbeing of all the stakeholders to complement their knowledge of the training programmes. Once achieved, the entire system would stimulate a ripple effect, enhancing the experience of the children at the heart of the MC program. This would be the start of a transformation process across the whole system. Doolittle and Jones [(9), p. 5] and Humphrey et al. [(10), p. 3] emphasise that SEL skills are closely linked to societal values. As seen in Figure 1, MC consciously incorporated the Measurement for Change system (M4C). M4C is an integrated monitoring, evaluating, and learning system that evaluates and strengthens the capacity to make decisions for effective sustainability [(11, 12), p. 1]. The system advocates for the process to be dynamic, inclusive, informative, interactive, and people-centred. MC used this approach so that the process of design and engagement could be contextualized and driven by the needs and values of the organisation. There was explicit intent to focus on all five aspirations throughout the process.

A core team of four members was created as the initial step. This team, with support from the subject experts, developed a Theory of Change (Supplementary Appendix 1), established internal consensus, and identified five key SEL constructs. These were accountability, integrity, respecting boundaries, empathy, and vulnerability. They were identified from our personal and collective experiences and meaning in our context Anziom et al. (13). An internal review of the culture of the organisation, at the beginning of the year, had also highlighted gaps in the identified indicators. These constructs also resonate with the clustering of competencies in emotional intelligence [(14), p. 611].

**Figure 2:**
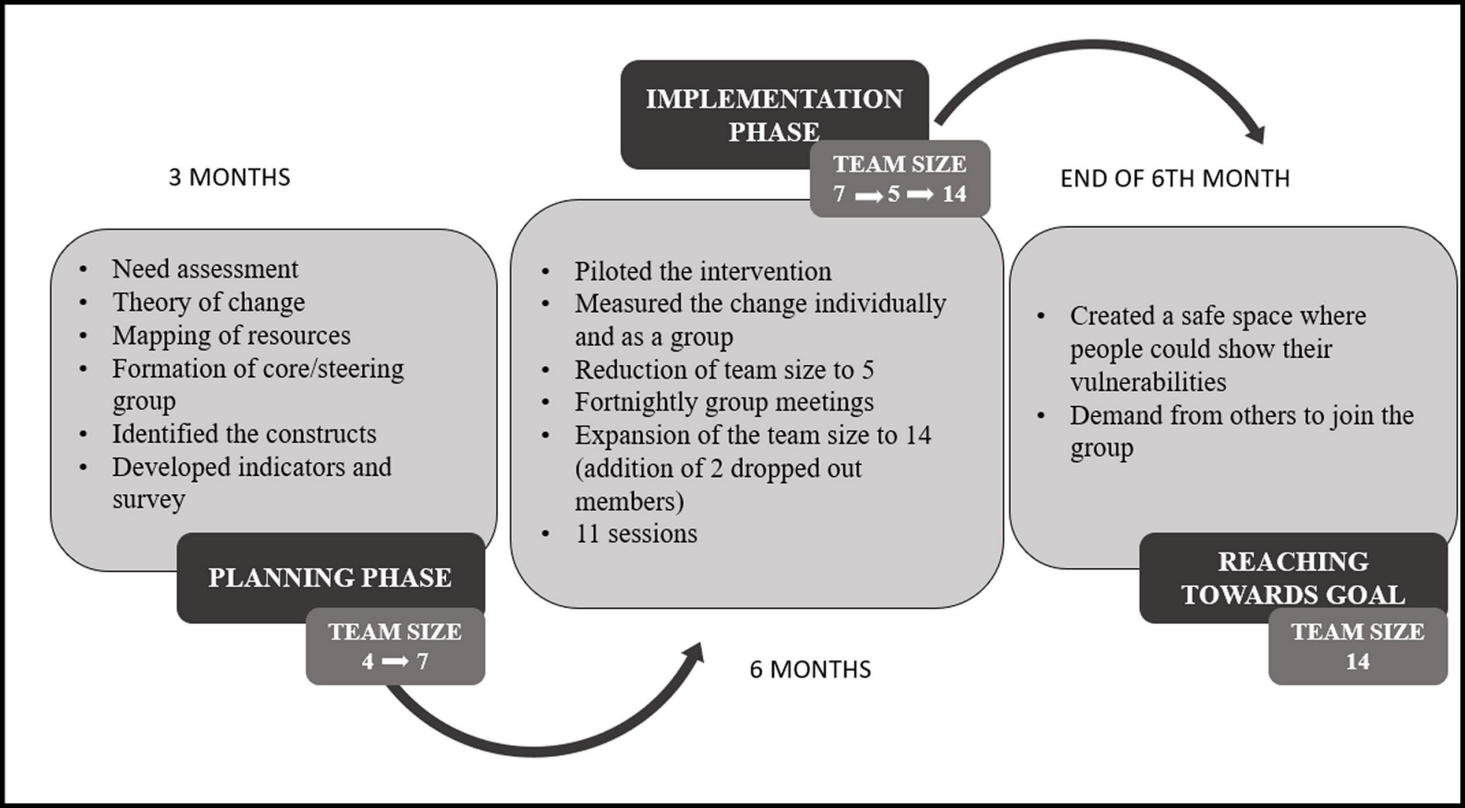
The process of change—development of a cell organizational framework.

Figure 2 shows the development of our SEL organisational framework. It shows the process of change over time, highlighting its responsive nature. The process started in July and ended in March. The planning phase was 2 months, and the implementation phase was 7 months. From the initial core team of four members, we expanded to 14. The size of the team grew from 3 to 9.4% of the total members in the organisation (150) by the end of the intervention. In the initial planning phase, core team members were identified based on the criteria that they should have had a prior understanding of SEL. The four members were part of MC’s implementation programmes from the onset, which included childcare services and capacity building programmes. However, there was an exception of one member who was from the fundraising and communication department. He was added as a core team member due to his zeal and passion for the subject. Three new members were invited to join this core team in the middle of the planning stage. These new members were directly working with the SEL curriculum and implementation of childcare services. This team started measuring themselves against the five constructs. During this process, the collective group identified that two of the seven members were unable to meet the requirements of the meeting times due to overcommitment on their part. A mutual agreement was reached for these two members to opt out temporarily and join the next intake of participants that would then form the expanded team. This led to a five-member core team at this point.

Each construct was defined by indicators made up of key behaviours that the team used to assess and monitor progress. The tracking was done after each session via a self-analytical survey. This was then translated to how the group was progressing as a whole. A copy of the final self-analytical survey can be found in Supplementary Appendix 2.

Reflective discussions and tracking of these indicators in the following 2 months highlighted the need to remove one construct, vulnerability, and condense the number of indicators being tracked. Vulnerability was seen to be an outcome achieved through changes in the other constructs. The group collectively realized that a vulnerable space would be created if all four constructs were achieved. Brené Brown’s study on vulnerability contributed to the group’s perspective on vulnerability. In the past, vulnerability was equated with weakness. However, today, it is seen as a crucial attribute of leadership. A recent study showed that individuals willing to show their vulnerabilities had higher emotional intelligence, were happier, more interpersonally skilled, and mentally tougher [(15), p. 1–2]. The team found fortnightly meetings more efficient, with a focused number of indicators to review and discuss. Another realization that came forth whilst discussing the achievements and shortcomings was that the interpretation of the indicators differed significantly. Consequently, close-ended statements with simplified language for all indicators were developed. The team also stated the addition of an underlying guiding principle to contextualize the framework of the monitoring and evaluation process. This was to track the team’s progress with the best interest of the organisation in mind, signifying the rights and responsibilities of all members. The continual interactive process was crucial in strengthening the monitoring and evaluation process, enhancing the reliability of the data collection. Details of the final four SEL constructs, with their respective indicators, can be found in Supplementary Appendix 3. The ranking and analysis of the results were recorded in an Excel sheet, allowing ongoing monitoring and evaluation of progress toward the identified goals.

In the implementation phase, nine more members from varied departments joined the team, including the two who had opted out earlier. This now made up a 14-member team. Each head of department nominated a minimum of one member from their department to join the group. Here, it is imperative to point out that the executive director of MC supported this intervention and was kept in the loop at every phase of the development. The objective was to reinforce the practice and broaden the environment that fosters the social and emotional wellbeing of all the stakeholders. We intentionally sought to include members of diverse religions, backgrounds, and gender. In India, the caste system still dictates much of life, including where you live, work, and who you socialize with Sahgal et al. [(16), p. 96–107]. Kaul [(17), p. 10–43] reminds us of the Indian experience in relation to contact theory. It states that the more time people from diverse backgrounds spend with each other, the more understanding and harmony increase. The intention of being inclusive was to strengthen the understanding, applicability, and reach of the intervention.

Each new member was taken through an exploration exercise. Reflective questions and scenarios were used to get a shared understanding of their existing levels and understanding of socio-emotional skills. The purpose of this was to build on and from their current understanding and to inform them how the following sessions would be designed. An orientation session detailed the SEL framework with the four constructs, their importance, and their role in driving the desired change. During this time, the team built a shared understanding of how every indicator is aligned with the four SEL constructs. There was an extensive discussion on how each SEL construct can be demonstrated amongst all the team members, as well as with the others in their respective professional departments. This was to ensure that there was a common understanding of all the indicators within each construct, assuring the alignment of measuring and practicing the social–emotional skills. The interactive nature of the sessions directed the choice of topics for the future weeks, ensuring individual responsibility. The objective here was the intentional application of positive social–emotional skills. Apart from using PowerPoint presentations, role plays, and videos to stimulate discussions on these topics, some unique situational questions were given to the participants to encourage reflective thinking while connecting such situations to their own personal life situations.

## Results

Data were collated from systematic observations in the workplaces and captured in notes during our group meetings, core group reflections, and surveys. We found that these methods of collecting, managing, and interpreting data were feasible in our context.

The five core team members collected data both during the 11 SEL sessions and from discussions with the wider team members during interactions within the workplace. Members shared how they have become more aware of analysing their own actions and their peers’ responses with reference to the SEL constructs. They were able to identify their mistakes, one of the indicators of integrity, and acknowledge them for themselves and within their group with the objective of rectifying it. The sharing of personal stories highlighted essential insights into personal reflective practice as well as providing opportunities to engage with one another’s learning.

Insight 1: One experience shared was about how a member could not ignore and move on while a child, living on the pavement, was getting beaten up by her mother. She stated that the change in her was that previously, she would not have gone on to interact with that parent and child. Having learned about the potential impact on brain functions from positive and negative adult–child interaction, she felt empowered to do so. This member was from the expanded team and was working in the accounts department.

Insight 2: Three members from the administration, finance, and front desk department, respectively, and also from the expanded team, shared that they had developed an interest in understanding why an individual reacts and responds in the way they do. They had started watching informative videos and reading articles to further forge an understanding of SEL independently.

During the focus on integrity, various tools, including meditation, journaling, and painting, were used to bring awareness. With a session focused on respecting boundaries, an emphasis was placed on practicing ‘Stop, Think and Act’ for conflicting and difficult situations. During the session, members reflected on a recent conflict situation where they experienced strong emotions, both positive and negative. They labelled their emotions and identified the reasons, however big or small, that contributed to that feeling.

Insight 3: One member had become aware of her daughter’s expressions and behaviour while scolding her. She reported instantly changing her own behaviour and intentionally managing the way she then spoke to her daughter. This member was working in the accounts department.

Insight 4: While sharing their responses, the members voiced that they were able to share their workplace issues within the group because they felt comfortable and assured that each member was striving to build a socially and emotionally sensitive approach. The sessions and space also helped them resolve conflicts with the fellow team members, keeping the shared vision at the core.

A disruption toward the end of the intervention, the third wave of COVID-19, replaced the in-person sessions with virtual ones. Further interruption was caused by the demand placed on the team members. The stretch between looking after sick family and attending other online operation meetings reflected in inconsistent attendance. However, the observation was that when members did join, they shared gratitude toward having this collaborative and safe space.

In addition, each member completed the self-analytical survey after each session. At the end of each session, the 14 surveys were analyzed by the core team. The team looked at how each member scored each indicator. The number of members who marked as having met the criteria (marking agreed or strongly agreed) was added and converted to a colour based on a rubric presented in Table 1. A rubric was designed to quantitatively represent the data, made up of three broad categories—red, yellow, and green (Table 1). When less than 40% of the team, that is, no more than five people, demonstrated the practice of the behaviour, the progress tab was marked red. Yellow indicates that 40–75% of people (between six to 10 members) were successful at practicing a behaviour, and green when over 75% of the people (11 or more members) demonstrated this practice. The practice of each behaviour was colour-coded 11 times, over the period of the 11 sessions. At the end of 11 sessions, the number of red, yellow, and green were added within each construct and converted into percentages. This is presented in Table 2.

**Table 1:** Rubric to show how the data were quantified.

Red (less than 40%)	Yellow (Between 40–75%)	Green (more than 75% people)
Less than or equal to 5 people	Between 6 and 10 people	11 or more people

**Table 2:** Progress of the constructs.

Constructs	Progress – percentage of the 3 broad categories over the 11 sessions		
	Red	Yellow	Green
Accountability	27%	52%	21%
Integrity	0%	33%	67%
Respecting boundaries	9%	52%	39%
Empathy	9%	47%	44%

Triangulation of the different sources of data enabled the team to evaluate the impact of the reflective and learning process.

Table 2 shows that not all four constructs were achieved at the same level. Integrity showed the most improvement. The most improved indicator within this construct was the ability to not discuss a disagreement or shortcoming concerning any colleague in their absence. Within the construct of empathy, the listening skill improved and remained constant after the initial two sessions.

## Discussion

The findings from the case study indicate that there was a positive shift in the practice of social–emotional skills for the team. The premise of the SEL-focused intervention trialled within a subset of the MC organisation arose from the need to promote a practice and environment that fostered social and emotional wellbeing. This was in response to addressing the gap between the organisation’s training program focusing on providing a responsive environment for the children and their own social and emotional wellbeing. According to Bicchieri (18), cited in Randolph et al. [(8), p. 250–262], the social field must also shift, and this happens only when there is collective action for social change and social sanctions to reinforce and maintain the desired changes. With the upskilling of individuals in modelling social–emotional skills, this was the start of the process of the desired system change. The design and application of the intervention framework was directly informed by the needs and values of MC, a core aspiration of M4C. This is also in line with that stated by Boyatzis et al. (19). He stated that an intentional change process must begin with a person wanting to change, and the Intentional Change Theory provides a framework to understand that in a sustainable way.

The reflective insights shared by the members highlighted intentional practice and awareness of their behaviours. The interest in learning more about SEL, as reported by three members, and the appreciation of the safe space created point toward being open to change. The findings also reflected an increase in the level of engagement with and empowerment by using the social–emotional skills Suleman et al. (20). Randolph et al. [(21), p. 207–211] confirmed, although it was in a school setting, that safe settings lead to building and sustaining positive learning environments. The shift here highlights the work of Salovey and Mayer [(22), p. 190] and Mayer and Salovey [(23), p. 433] on emotional intelligence. This is the ability to be aware of emotions and to be able to regulate them toward oneself as well as others (24).

The period of study also showed the team’s strength in the construct of integrity. Suleman et al. (20) showed that emotional intelligence is a central variable in contributing toward an organisation’s productivity, of which integrity is a core construct. Going forward, the team can continue to practice the way it does to sustain this construct. Data on the other three constructs, accountability, respecting boundaries, and empathy, suggest more integration in practice. Having said this, one of the reasons why accountability was impacted was the lockdown due to COVID-19. One behaviour under this construct was practiced in less than 40% of the team for a significant chunk of time. The indicator was to take responsibility to attend the meeting on time and, in case of absence, make sure to inform and share the needful information with the team. However, feedback from the group, when they met virtually, continued to highlight the appreciation of the safe space created.

Whilst the data identified integrity as the team’s strength, the individual members exhibited trust as central; the experience of being in a safe space is proposed to have reflected in the commitment toward the practice of integrity. Another observation was that although the participants engaged positively during the sessions, they did not necessarily always demonstrate the behaviours from the four constructs consistently in the wider systems they were part of. This could be suggestive of various reasons. First, individual safety and confidence to roll out similar conversations with their wider teams. The other could be the difference in the physical environments where the meetings were held. It would be interesting to observe the participant’s confidence over a longer time or the ‘action confidence’ [(25), p. 154134462094081]. This is the participants’ relationship to taking action and the way this changed over the course of their participation in the learning process. In aspiring toward a transformative change, the data tracked over time would be informative in making decisions on the next stage, an important element of the M4C process.

The continual reflective process when designing the framework with the constructs was made inclusive. Feedback from the core team members and establishing a shared understanding of what indicators to settle for and the details within each highlighted the interactive nature of the exercise. The willingness to discuss, modify, and revisit the framework over the initial weeks pointed to the dynamic nature of how feedback was used to come up with the final list of constructs and its respective indicators. Whereas the team acknowledged the limitation of rating individual progress on each indicator when observed outside of the session setting, the practice of discussing the observations as a team must be noted. Every member’s input was taken, leading to a positive engagement, as confirmed by the members.

Klitkou et al. (2) emphasised the benefits of combining qualitative and quantitative methods to gain a richer understanding of interconnected social practices. Whilst this can be a time-consuming exercise, it is imperative to set the stage before rolling out the intervention to the larger ecosystem. The adoption of an interactive approach in monitoring and evaluating the data provided a framework that was reflective of the core team’s needs and context. The ability to participate in this decision-making process enhances the intervention to be more sustainable (12).

Whilst every effort was made to make the team of 14 members inclusive, having members from varied departments, it was noted that some of the senior management team prioritized other operational meetings during the session times. This gave the perception of the intervention being less important. Chastukhina (26) found that emotionally intelligent leaders apply their social capacities to influence and motivate others and ensure long-lasting relationships with their teams. Since the inclusion through diversity in caste systems, religious practices, gender, and departmental systems was considered in the initial intervention cycle, it is proposed that the future cycle includes more members from the senior leadership team. The objective of this would be to enable systemic change and the aspiration of being inclusive to be met [(11), p. 1]. This would also facilitate the systemic practice of socio-emotional skills across the organisation, heightening its impact and reach to the stakeholders outside of the organisation. Feedback from the current case study already suggested a change in the quality of conversations and, therefore, the dynamics of relationships with all those they interacted with.

The results from the 11-week intervention already show a shift in the chosen SEL constructs, some more and some less. The time taken to realize this shift at the level of creating a responsive environment for the children may take time. However, the importance of successfully establishing this culture change within the ecosystem will not only show us what can change but, most importantly, how we can consciously create a sustainable change. Monitoring and evaluating the intervention with the M4C approach allowed the team to continually reflect on the why and how of the measurement. The current case study was limited by its sample size, but it was important to establish a framework and a process of integrating SEL into organisational practice at a smaller scale at first. As Druskat and Wolff [(5), p. 81] highlighted, creating an upward, self-reinforcing spiral of trust, group identity, and group efficacy requires a team atmosphere in which the norms of the group build its emotional capacity. This group then adopts an ambassadorial role in developing the capacities and confidence of the other members of the ecosystem. For our next phase, the plan is to build monthly 2-h sessions for the entire office staff, which make up 40% of the organisation. This includes members from the team of trainers and middle and senior management (refer to Figure 1). These meetings would follow the same approach to engage members in socio-emotional activities with the objective of enhancing their capacity. This newly trained team would then build the capacity of the frontline workers within their scheduled monthly sessions in the office. It is hoped that the journey of transitioning to scale will give further insights to the team that will continue to inform the decision-making process and be inclusive, people-centred, and interactive. Our recommendation from this case study is to use our process. However, it is important to use local values embedded in one’s context and establish needs to drive it toward a sustainable change.

## Data availability statement

The original contributions presented in the study are included in the article/Supplementary material, further inquiries can be directed to the corresponding author.

## Ethics statement

Written informed consent was obtained from the individual(s) for the publication of any potentially identifiable images or data included in this article.

## Author contributions

All authors listed have made a substantial, direct, and intellectual contribution to the work and approved it for publication.
