# Supplementary figures and images for: Measuring for change/Mobile Creches
Source: Front Public Health. 2024 Jan 29;11:1165642. doi: 10.3389/fpubh.2023.1165642 (PMC10859431; doi:10.3389/fpubh.2023.1165642)

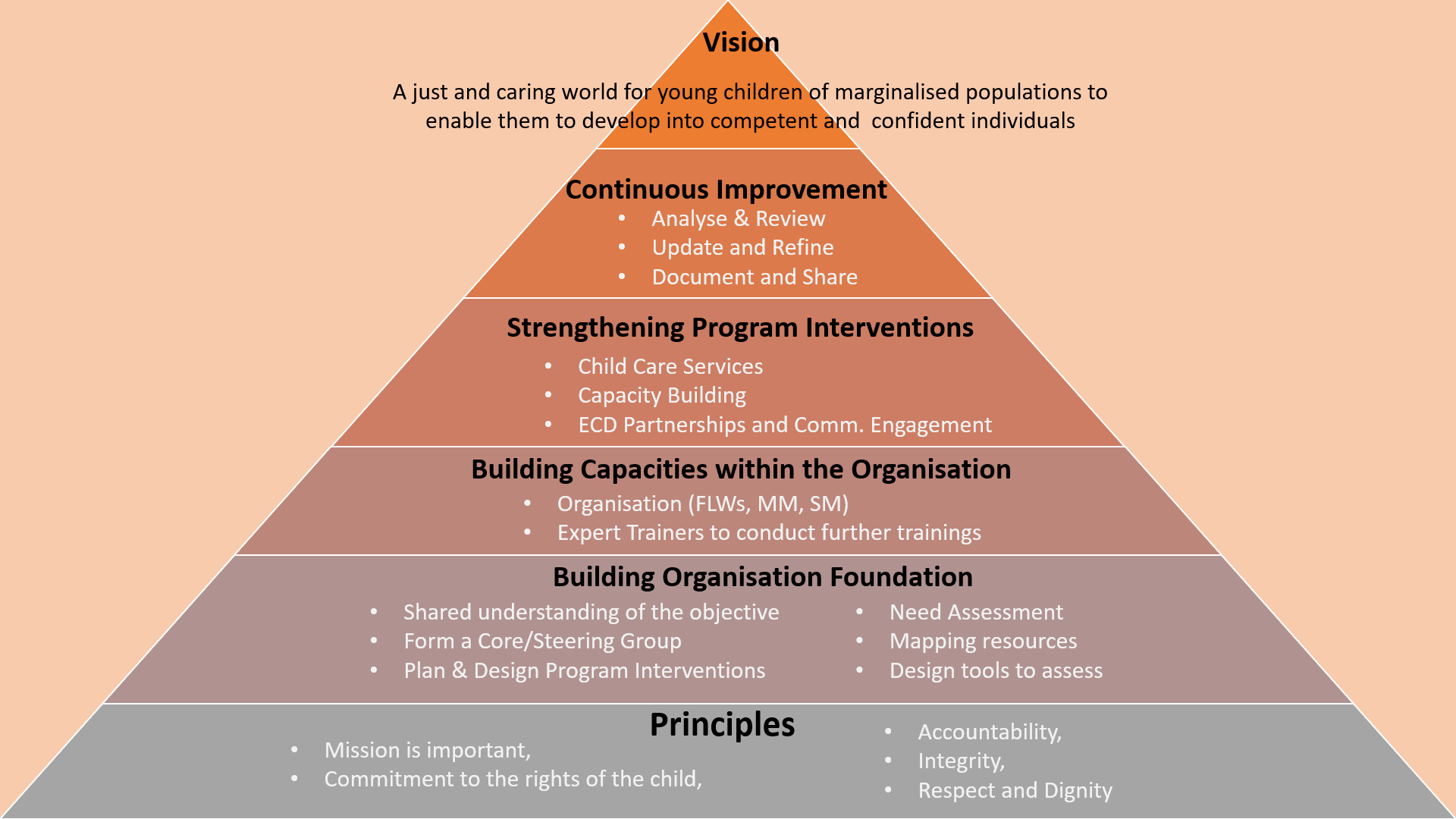

Supplement: Supplementary file 1 [file Image_1.png]
