# Supplementary material for: Measuring for change/Mobile Creches
Source: Front Public Health. 2024 Jan 29;11:1165642. doi: 10.3389/fpubh.2023.1165642 (PMC10859431; doi:10.3389/fpubh.2023.1165642)
Supplement: Supplementary file 2 [file Image_2.pdf]

SEL Core Team- Indicators Assessment

Accountability

* Required

1. Are we all updating about the tasks we commit to, keeping in mind the timelines? *

Mark only one oval.

- Strongly Disagree
- Disagree
- Agree
- Strongly Agree

2. Are we all asking for updates, about each task, among ourselves? *

Mark only one oval.

- Strongly Disagree
- Disagree
- Agree
- Strongly Agree

3. Do we attend meetings on time or inform the team, in case of absence, and share the needful information with the team *

Mark only one oval.

- Strongly Disagree
- Disagree
- Agree
- Strongly Agree

4. Are we being mindful about the competencies and limitations of each one of us, and asking/seeking support in upscaling respective person to meet the required task/goal at hand *

Mark only one oval.

- Strongly Disagree
- Disagree
- Agree
- Strongly Agree

Integrity

5. Are we giving honest feedback to the group with an intention to improve the quality of work? *

Mark only one oval.

- Strongly Disagree
- Disagree
- Agree
- Strongly Agree

6. Are we all taking responsibility for our respective shortcomings? *

Mark only one oval.

- Strongly disagree
- Disagree
- Agree
- Strongly Agree

7. Are we taking initiative to resolve conflicts in a constructive manner? (Open dialogues about disagreements, misunderstandings/ bringing clarity) *

Mark only one oval.

- Strongly Disagree
- Disagree
- Agree
- Strongly Agree

8. Are we discussing a disagreement/ shortcoming concerning any of our team member in his/her absence, with an intention to malign? *

Mark only one oval.

- Strongly disagree
- Disagree
- Agree
- Strongly agree

Respecting Boundaries

9. Are we all being mindful about not making personal remarks (compliment/ confrontation)? *

Mark only one oval.

- Strongly Disagree
- Disagree
- Agree
- Strongly Agree

10. Are we clearly communicating/ asking what is comfortable with each of us? *

Mark only one oval.

- Strongly Disagree
- Disagree
- Agree
- Strongly Agree

11. Are we sending a text/ message before calling each other, beyond working hours? *

Mark only one oval.

- Strongly Disagree
- Disagree
- Agree
- Strongly Agree

Empathy

12. Do we regularly keep a check on how everyone is feeling? *

Mark only one oval.

- Strongly Disagree
- Disagree
- Agree
- Strongly Agree

13. Do we take follow ups regarding any of our team member's concern? *

Mark only one oval.

- Strongly Disagree
- Disagree
- Agree
- Strongly Agree

14. Do we listen without judgement/ pre- conceived notion about each other, to understand the other one's perspective? *

Mark only one oval.

- Strongly Disagree
- Disagree
- Agree
- Strongly Agree

15. Do we acknowledge and appreciate sharing/contribution of others? *

Mark only one oval.

- Strongly Disagree
- Disagree
- Agree
- Strongly Agree

16. Are we condemning each other for our mistakes? *

Mark only one oval.

- Strongly Disagree
- Disagree
- Agree
- Strongly Agree

This content is neither created nor endorsed by Google.

Google Forms
