# Supplementary material for: Measuring for change/Mobile Creches
Source: Front Public Health. 2024 Jan 29;11:1165642. doi: 10.3389/fpubh.2023.1165642 (PMC10859431; doi:10.3389/fpubh.2023.1165642)
Supplement: Supplementary file 3 [file Image_3.pdf]

S.No	Week/Date	Focus	Session
1.	22/11/21	Orientation on SEL	Orientation and introduction on SEL intervention. What is it and why are we doing this?
2.	23/12/21	Neuroscience of SEL	Presented Dr. Dan Siegel hand model of brain. Discussion on how the brain functions and what are the core SEL competencies and how are they related to SEL indicators
4.	30/12/21	Self Awareness	Discussion on self awareness reflections after a video was shown on SA by Dr. Nicole Le Pera
5.	6/1/22	Self-Awareness and Self-management	Self awareness reflection tools and meditation activity
6.	12/1/22	Self-Awareness, Self-management, Decision Making	Discussion on self-management and 'Stop-Think-Act' practice.
7.	20/1/22	Self – Awareness	Discussion- if and how the past one month intervention has created an impact in how each one responds in any situation and feedback for the group
8.	27/1/22	Social Awareness	Pairing 2 members and sharing perceptions- by looking at them (their mood, thoughts, gender etc) Discussion on Social Awareness and its importance through jam board and video activities
9.	17/2/22	Self-Awareness, Self-management, Decision making	Open discussion on 'What do we need to achieve?' (Goals) and tracking back to "How do we achieve?" (Action points)
10.	24/2/22	Social- Awareness	Role play by team members for demonstrating and analysing how empathy is different in varying situations
11.	3/3/22	Social- Awareness	Role plays and discussions on empathy and how we can demonstrate them in professional sphere
